# Differences in Mate Pairings of Hatchery- and Natural-Origin Coho Salmon Inferred from Offspring Genotypes

**DOI:** 10.1093/iob/obab020

**Published:** 2021-08-14

**Authors:** H L Auld, D P Jacobson, A C Rhodes, M A Banks

**Affiliations:** Department of Fisheries, Wildlife, and Conservation Sciences, Oregon State University, Corvallis, OR 97331, USA; Coastal Oregon Marine Experiment Station, Hatfield Marine Science Center, Oregon State University, Newport, OR 97365, USA; Department of Fisheries, Wildlife, and Conservation Sciences, Oregon State University, Corvallis, OR 97331, USA; Coastal Oregon Marine Experiment Station, Hatfield Marine Science Center, Oregon State University, Newport, OR 97365, USA; Coastal Oregon Marine Experiment Station, Hatfield Marine Science Center, Oregon State University, Newport, OR 97365, USA; National Center for Biotechnology Information at the National Library of Medicine, National Institutes of Health, Bethesda, MD 20894, USA; Department of Fisheries, Wildlife, and Conservation Sciences, Oregon State University, Corvallis, OR 97331, USA; Coastal Oregon Marine Experiment Station, Hatfield Marine Science Center, Oregon State University, Newport, OR 97365, USA

## Abstract

Captive breeding can affect how sexual selection acts on subsequent generations. One context where this is important is in fish hatcheries. In many salmon hatcheries, spawning is controlled artificially and offspring are reared in captivity before release into the wild. While previous studies have suggested that hatchery- and natural-origin fish may make different mate choice decisions, it remains to be determined how hatchery fish may be making different mate choice decisions compared with natural-origin fish at a genetic level. Using genotyping-by-sequencing, we identify single-nucleotide polymorphisms (SNPs) associated with variation in mate pairings from a natural context involving hatchery- and natural-origin coho salmon (*Oncorhynchus kisutch*). In both natural-origin and hatchery mate pairs, we observed more SNPs with negative assortment than positive assortment. However, only 3% of the negative assortment SNPs were shared between the two mating groups, and 1% of the positive assortment SNPs were shared between the two mating groups, indicating divergence in mating cues between wild and hatchery-raised salmon. These findings shed light on mate choice in general and may have important implications in the conservation management of species as well as for improving other captive breeding scenarios. There remains much to discover about mate choice in salmon and research described here reflects our intent to test the potential of ongoing advances in population genomics to develop new hatchery practices that may improve the performance of hatchery offspring, lessening the differences and thus potential impacts upon wild stocks.

## Introduction

Recent evidence suggests that captive breeding can affect mating preferences among offspring (Slade et al. 2014) and that individuals who choose their mates often have greater mating success than those whose mate choice is constrained (e.g., giant panda—Martin-Wintle et al. 2015; house mice—Drickamer et al. 2000; Gowaty et al. 2003; *Drosophila*—Anderson et al. 2007; mallards—Bluhm and Gowaty 2004). There is also evidence that captive-bred individuals have lower reproductive success relative to their natural-origin counterparts even when they are able to make their own mate choice decisions in the wild (e.g., Berejikian et al. 2001; Araki et al. 2008, 2009; Thériault et al. 2011; Whitcomb et al. 2014; see Farquharson et al. 2018 for a review; Jonsson et al. 2019). The reasons for the observed reduction in reproductive success are not well understood, but it should be noted that animals bred and raised in captivity often differ from their natural-origin counterparts in behavioral and morphological characteristics (e.g., see O'Regan and Kitchener 2005 for a review in mammals; Blanchet et al. 2008). The observed difference in reproductive success between individuals who choose their mates and those who do not may in part be attributable to the inability of humans to identify optimal mating pairs, in a controlled breeding scenario used for conservation management, due to a lack of information on the factors that affect mate choice and reproductive success in a wild system or due to the anthropomorphic bias toward human sensory modalities. Understanding traits underlying mate choice in the wild can help resolve theoretical questions and improve breeding programs (Quader 2005).

One group of animals for which artificial breeding occurs frequently is Pacific salmon (*Oncorhynchus*). Pacific salmon are a culturally and economically important species in many places in the northern hemisphere. Unfortunately, salmon stocks have been declining as the result of human activity that increases coastal pollution and habitat destruction in traditional salmon production regions. In an attempt to supplement declining salmon runs, stocks have been bred in fish hatcheries on the western coast of North America since the 19th century. While there are many positive aspects to improvements in hatchery practice that have occurred over the last two centuries, there remain some concerns about these salmon hatcheries including, but not limited to, the observed reduction in reproductive success of hatchery fish (Whitcomb et al. 2014; Neff et al. 2015). It has been suggested that some of the observed reduction in reproductive success may be related to the absence of mate choice or sexual selection in parental generations (Neff et al. 2011; Thériault et al. 2011; Chargé et al. 2014).

Coho salmon (*Oncorhynchus kisutch*) is a culturally, ecologically and economically important species with strong commercial and recreational fisheries on the west coast of North America, Alaska, and Russia. Coho are anadromous and semelparous. Adult males and females as well as precocious jacks, which are males who sexually mature a year earlier than other males and females, return to their natal sites to reproduce. The semelparous nature of their mating system and their fidelity to spawning grounds facilitate measurement of overall lifetime reproductive success. Both males (Sargent et al. 1986) and females (Berejikian et al. 1997) can exhibit mate choice. Males can differentially allocate reproductive effort by means of courtship and intrasexual competition toward certain females over others. Females can exhibit mate choice by delaying release of eggs until in the presence of preferred mates. Moreover, while we are unaware of any studies on gamete-mediated mate choice in coho salmon, there is evidence of gamete-mediated mate choice in other salmonid species such as Chinook (*Oncorhynchus tshawytscha*) (Rosengrave et al. 2016) and Atlantic salmon (*Salmo salar*) (Yeates et al. 2009).

Here, we use a previously established pedigree of both hatchery- and natural-origin coho salmon from the Umpqua River in southern Oregon (Thériault et al. 2011; Whitcomb et al. 2014) and genotyping-by-sequencing (GBS) (Elshire et al. 2011) to identify single-nucleotide polymorphisms (SNPs) associated with variation in mate choice between hatchery- and natural-origin fish. While GBS does not sequence the entire genome, it sequences a reduced representation of the genome that can identify genetic variants throughout. Most studies that have incorporated genetic compatibility into mate choice studies have focused on immune-relevant major histocompatibility complex (MHC) alleles and/or overall genome-wide similarity or heterozygosity (e.g., Roberts and Gosling 2003; Forsberg et al. 2007; Kempenaers 2007; see Fukamachi et al. 2009 for an exception; O'Malley et al. 2015). The exploratory genome-wide approach used in this study allows us to evaluate a more general array of genes that may be relevant to mate choice in coho salmon.

In this study, we test for different patterns of nonrandom mating in the offspring (F1) of controlled crosses that were released into the wild and of natural-origin fish. The controlled crosses involved both wild- and hatchery-derived parents. We used reduced-representation SNP data to test hypotheses of positive vs. negative assortment of F1 mate pairs that were the product of wild parents or hatchery parents. Lastly, we used a reference genome to annotate subsets of positively and negatively assorting SNPs.

## Methods

### Experimental approach

**Fig. 1:**
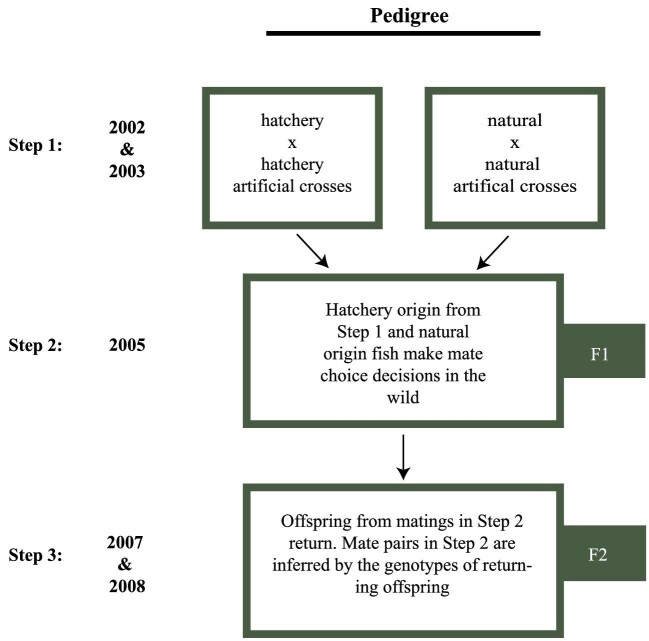
Sampling design of the study. Initial crosses were performed on hatchery- and natural-origin fish in 2002 and 2003. The offspring from those crosses (F1) and the offspring of naturally mating fish returned from the ocean in 2005. Finally, in 2007 and 2008, F2 offspring from the 2005 matings returned from the ocean. Mate pairs in 2005 were inferred from the genetics of the returning offspring.

Our approach takes advantage of a previous set of controlled crosses with hatchery-raised and wild fish, natal spawning behavior, and sampling and genotyping over two generations (Fig. 1). Crosses were conducted in 2002 and 2003 by staff biologists of the Oregon Department of Fisheries and Wildlife who collected returning coho salmon in southern Oregon's Umpqua River. Based on the presence or absence of an adipose fin, they identified the returns as either of hatchery- (absent) or natural-origin (present) and performed single mate crosses (Fig. 1, Step 1). Crosses consisted of either hatchery × hatchery (*n* = 100 in 2002 and 2003) or natural-origin × natural-origin fish (*n* = 100 in 2002 and 102 in 2003). All crosses had unique parents. The resultant offspring were reared in the hatchery and then released into a tributary of the Umpqua (Calapooya Creek) either as fry or as 1-year smolts. After a year in freshwater, coho salmon migrate to sea for 2 years before returning as 3-year-old adults. Some males sexually mature after only a year at sea and return to their natal streams as 2-year-old jacks.

The F1 of these crosses were sampled in 2005. Fish returning to Calapooya Creek (43°24′31.77N, 123°09′42.52W) were caught in a fish trap at the base of Nonpareil Dam on Calapooya Creek. Returning fish included offspring (F1) from the mate crosses performed in 2002 (adults) and 2003 (jacks) as well as the offspring of any wild matings in those years (Fig. 1, Step 2, F1) as determined by parentage analysis (Moyer et al. 2007, and see the next paragraph). Standard length and return date of each fish were recorded and a fin clip taken for genetic identification. Individuals were then released above the dam and allowed to participate in natural mating events wherein mate choice would have occurred. In 2007 and 2008, the offspring (F2) produced from these matings were again sampled from Calapooya Creek (Fig. 1, Step 3, F2). Upon return, standard length and return date of each fish were recorded and a fin clip taken for genetic identification.

The parentage of the F1 (2005) and F2 (2007 and 2008) generation (Fig. 1) was determined from a previously constructed pedigree (Moyer et al. 2007; Thériault et al. 2010, 2011; Whitcomb et al. 2014). Briefly, Moyer et al. (2007) used a maximum likelihood approach to assign F1 returns to parents from hatchery crosses made in 2002 and 2003 (Fig. 1, Step 1) using the software PAPA 2.0 (Duchesne et al. 2002). Next, the genetic identity of parents mating in the wild (Fig. 1, Step 2) and their returning offspring (Fig. 1, Step 3) was determined by Thériault et al. (2010) with a maximum likelihood and exclusion approach using PASOS 1.0 software (Duchesne et al. 2005). Using simulations, Thériault et al. (2011) determined that the percentage of fish that were assigned to the correct parents was 96%. Using these multigenerational data along with additional data, Moyer et al. (2007) first identified family correlated survival of two separate hatchery stocks (multigeneration brood stock and single-generation brood stock collected from the North Umpqua River). Next, Thériault et al. (2011) used this established pedigree to identify lower reproductive success in hatchery-origin fish that were released as unfed fry compared with natural-origin fish even when they are able to mate in the wild. This result suggested that differences in mate choice between hatchery and wild fish may result in the observed reduction in reproductive success, and are the focus of the present paper. To identify genetic loci that play a role in coho mate choice, we sequenced the F1 generation (Fig. 1) from the previous pedigree using GBS (Thériault et al. 2011) and compared the genetic combinations of mate pairs of both hatchery- and natural-origin to what would be expected at random.

### GBS and SNP discovery

DNA was extracted from samples of the F1 generation using a Qiagen (QIANGEN, Inc., Valencia, CA, USA) kit as per the manufacturer's instructions. Subsequent GBS was performed at the Center for Genome Research and Biocomputing at Oregon State University. Extracted DNA was quantified using dsDNA fluorophore and tested to be of suitable molecular weight for analysis and any samples that did not contain enough DNA for GBS were discarded (*n* = 36). Minimum concentration of starting DNA was 4.00 ng/μL. The amount of DNA across samples was normalized to 100 ng. For the remaining samples, DNA was digested using an enzyme combination of PSTI (6-cutter) and MSPI (4-cutter). After fragmentation, a unique barcode for identification of individual samples and a common adapter were ligated to the DNA fragments before pooling. Amplification of fragments and sequencing (150 base pairs [bp] paired-end reads) was performed using Illumina HiSeq 3000 (Illumina Inc., San Diego, CA, USA). Read quality was assessed using FastQC.

Next, raw reads were processed through the STACKS v. 2.41 pipeline (Rochette et al. 2019). Reads were first trimmed to 134 bp and demultiplexed using *process_radtags* v. 2.41. For *process rad_tags*, the minimum phred score was 10 and the number of mismatches allowed in the adapter sequence was 2. After demultiplexing, reads were aligned to the coho reference genome (GenBank: MPKV00000000.1) using BWA v. 0.7.12 (Li and Durbin 2009).

Alignments were made to the indexed reference genome using the *bwa mem* algorithm and shorter split hits were marked as secondary. *G stacks v. 2.41* was used to assemble the loci and *Stacks* populations was used to call variant SNP loci. The minimum number of populations (*p*) a locus must be present in to process a locus was 1 and the minimum percentage of individuals in a population (*r*) required to process the locus was 0.8. Data analysis was restricted to the first SNP per locus. We did not include in the analysis individuals for which the number of reads in the bam file was less than 1,000,000 reads (40 individuals). This left 814 individuals for further analyses.

After SNPs were called, we used a modified version of the program of McKinney et al. (2017) to identify paralogs (Ortiz 2018). The parameters for the program were as follows: the maximum limit for heterozygosity was 0.6, the minimum read ratio deviation was −5, and the maximum read ratio deviation was 7. We then reran populations with a blacklist of identified paralogs. After this run of populations, there were 25,658 sites.

Next, we used *vcftools* to further filter data. First, we removed 36 individuals that had more than 20% missing data. We excluded 10,204 sites where the mean depth was less than 5 or exceeded 30 over all individuals. Finally, three sites where the proportion of missing data was greater than 80% were excluded. After filtering, 15,451 sites remained. No sites were removed for being out of Hardy–Weinberg equilibrium (see Fig. S1). Linkage disequilibrium was calculated and plotted with PopLDdecay (Zhang et al. 2019).

### Mate selection

To ascertain whether individuals were mating nonrandomly at each given SNP, and whether an individual's recent ancestry (i.e., hatchery or natural origin) affected mate choice pairings relative to each SNP, we first converted genotype information of each individual to minor allele dosage, which is the probability that any allele selected at random will be the minor allele. For any given locus, this probability was assigned 0 if an individual did not possess a minor allele, 1 if they had one minor allele, and 2 if they had two minor alleles. Next, we compiled all unique mating combinations of the F1 generation (*n* = 516). Thériault et al. (2011) used paternity analysis of the F2 generation to identify mating combinations of the F1 generation. If we were unable to successfully sequence one or both individuals in each previously identified mate pair using GBS, the entire pair was removed from our analysis. In all, we identified 142 hatchery male × hatchery female, 152 natural-origin male × natural-origin female, 118 natural-origin male × hatchery female, and 104 hatchery male × natural-origin female mating pairs in our genetic data. Because not all individuals were able to be genotyped at each SNP, sample sizes at each SNP may differ. We determined genotypic similarity at each locus, following Forsberg et al. (2007) and Whitcomb et al. (2014). We identified whether the mate pairs shared two (e.g., AA and AA or Aa and Aa), one (e.g., Aa and AA), or no (e.g., AA and aa) alleles at each genotyped locus that was of high enough quality to make it through filtering. If a mate pair shared two alleles at a given locus, they were assigned 2; if they shared only one allele, they were assigned 1; and if they shared no alleles, they were assigned 0. This process was repeated for each mate pair at each locus.

#### Within group comparison to random expectation

We used permutations to ascertain whether mate pairings at each identified SNP differed from random. Permutations were carried out using a custom R script and the packages purrr (Henry and Wickham 2018), Dplyr (Wickham et al. 2018), and tidyr (Wickham and Henry 2018) (see Coho Mate Choice Code in the supplementary material for detailed code). The null model was generated by randomly assigning a male from the dataset as a mate partner for each female in the dataset. We created 50,000 pairs for each SNP that diagnosed either natural origin–natural origin or hatchery origin–hatchery origin mate pairings. Because we used the genotypes of the individuals that were in the original dataset, the expected frequencies were generated from allele frequencies at each SNP. The percentile of the observed mean of shared alleles was calculated from the distribution of the mean number of shared alleles in mate pairs generated in each of the 50,000 pairs (see Coho Mate Choice Code in the supplementary material). If the percentile of the observed mean was below 0.025 or above 0.975, we identified those loci as demonstrating the strongest deviation from the null model. We then compared the sets of SNPs that showed patterns of positive and negative assortment across these two mating categories. All statistical analyses were completed in R (R Core Team 2018).

#### SNP annotation

Finally, we used the coho reference genome at the National Center for Biotechnology Information (NCBI) (GenBank: MPKV00000000.1) to identify any genes associated with SNPs related to different mating patterns between hatchery- and natural-origin fish. We only annotated genes that had a SNP within the gene itself. We did not identify upstream or downstream genes.

## Results

### SNP calling

Overall, there were 15,451 SNPs identified over the 30 chromosomes and unplaced scaffolds (Table 1). The identified SNPs were relatively equally dispersed across chromosomes. However, the average frequency of SNPs/chromosome length (bp) was <0.001 (Table 1). The heterozygote miscall rate as estimated by *whoa* (Anderson 2019) was 0.626% (Fig. S3).

**Table 1:** The length of the genome, length of individual chromosomes measured in base pairs (bp), and number of bp in unplaced scaffolds. Identified SNPs are included as absolute numbers and relative to the length of each chromosome.

Chromosome	Length (bp)	Number of SNPs	% of genome with identified SNP
Total	2,369,915,580	15,451	0.000652
1	67,385,680	500	0.000742
2	74,323,923	543	0.000731
3	70,097,442	537	0.000766
4	79,859,423	652	0.000816
5	71,784,841	572	0.000797
6	76,709,058	607	0.000791
7	50,431,327	411	0.000815
8	67,521,836	532	0.000788
9	39,647,964	285	0.000719
10	65,230,829	496	0.00076
11	79,463,822	599	0.000754
12	51,280,603	366	0.000714
13	66,844,158	470	0.000703
14	71,386,482	537	0.000752
15	67,073,436	526	0.000784
16	33,801,654	266	0.000787
17	75,567,179	558	0.000738
18	66,477,694	468	0.000704
19	54,998,362	385	0.0007
20	40,560,786	340	0.000838
21	34,965,661	276	0.000789
22	55,596,650	472	0.000849
23	42,348,054	299	0.000706
24	39,320,843	299	0.00076
25	33,789,546	273	0.000808
26	43,500,090	342	0.000786
27	38,560,861	314	0.000814
28	47,366,153	379	0.0008
29	38,425,108	312	0.000812
30	42,261,227	334	0.00079
Unplaced	683,334,888	2501	0.000366

### Within group comparison to random expectation

**Fig. 2:**
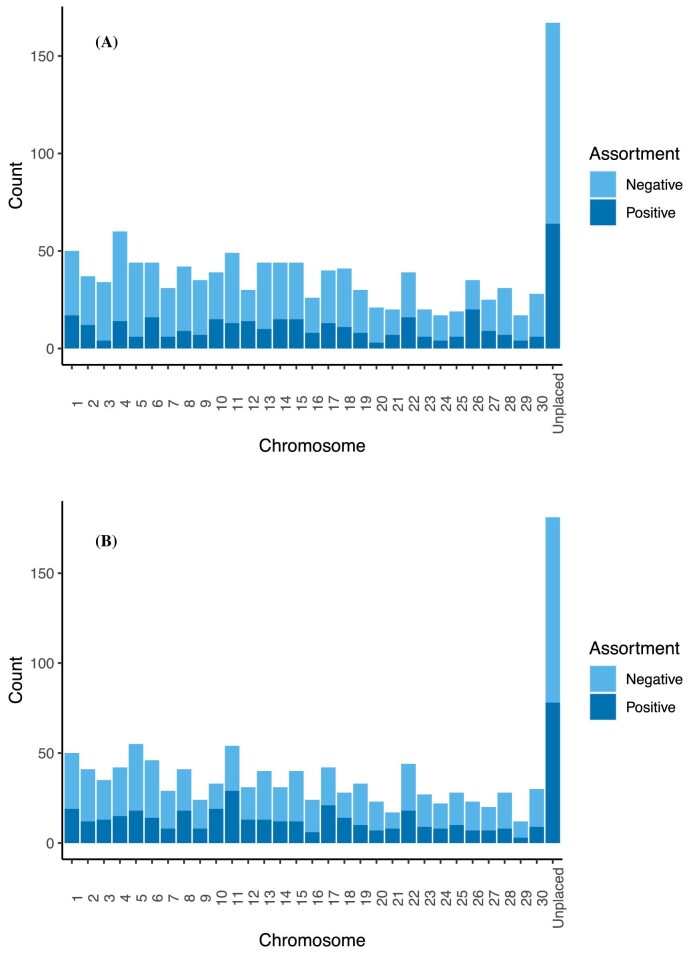
**(A)** Number of identified SNPs wherein natural-origin fish demonstrated either positive or negative mate assortment. Negative assortment was defined as when the percentile of the number of shared alleles in mate pairings was less than 0.025 in the distribution of all permutated means for that SNP. Positive assortment was defined as when the percentile of the observed mean number of shared alleles in mate pairings was greater than 0.975 in the distribution of all permutated means for that SNP. In all, there were 152 natural-origin × natural-origin matings; however, because not all individuals were able to be genotyped at each SNP, sample sizes at each SNP may differ. **(B)** Number of identified SNPs wherein hatchery-origin fish demonstrated either positive or negative mate assortment. Negative assortment was defined as when the percentile of the observed mean number of shared alleles in mate pairings was less than 0.025 in the distribution of all permutated means for that SNP. Positive assortment was defined as when the percentile of the observed mean number of shared alleles in mate pairings was greater than 0.975 in the distribution of all permutated means for that SNP. In all, there were 142 hatchery-origin × hatchery-origin matings; however, because not all individuals were able to be genotyped at each SNP, sample sizes at each SNP may differ.

In general, for both kinds of mate pairs (natural-origin vs. hatchery-origin), there were more SNPs that showed negative assortment than positive assortment, and there were relatively few SNPs shared between categories of mate pairs (Table 2). Positive and negative assortment was found at SNPs on every chromosome with similar frequency (Fig. 2).

**Table 2:** The number of positively or negatively assorted SNPs categorized by mating pairs and direction of deviation from random mating

Category	Negative assortment	Positive assortment
Wild parents	838	365
Hatchery parents	728	446
Shared	50 (3.19%)	11 (1.36%)

For mate pairs of natural origin, there were 838 SNPs where the average number of shared alleles among mate pairs was less than the 2.5th percentile of the null model, consistent with a greater degree of disassortative mating than would be expected by random mating (Fig. 2A; see Coho Mate Choice Gene Annotations in the supplementary material). In contrast, there were fewer SNPs (365) where the average number of shared alleles was greater than the 97.5th percentile of the null model, consistent with assortative mating at these loci (Fig. 2A; see Coho Mate Choice Gene Annotations in the supplementary material).

For mate pairs of hatchery origin, there were 728 SNPs where the average number of shared alleles among mate pairs was less than the 2.5th percentile of the null model, consistent with a greater degree of disassortative mating than would be expected by random mating (Fig. 2B; see Coho Mate Choice Gene Annotations in the supplementary material). In contrast, there were fewer SNPs (446) where the average number of shared alleles was equal to or greater than the 97.5th percentile of the null model, consistent with assortative mating at these loci (Fig. 2B; see Coho Mate Choice Gene Annotations in the supplementary material). While the number of SNPs demonstrating positive and negative assortment is similar between hatchery- and natural-origin fish, the SNPs demonstrating those patterns are not the same in each category. For example, if natural-origin mate pairs demonstrate positive assortment at a given SNP, it is unlikely that hatchery-origin mate pairs will demonstrate positive assortment at the same SNP. Hatchery matings at 61 SNPs demonstrated similar mating patterns to natural-origin mate pairs (i.e., they demonstrated more positive or negative assortment among mate partners than would be expected at random in the same direction as natural-origin pairs) and at 39 SNPs exhibited opposite mating patterns to natural-origin pairs (i.e., they demonstrated more positive or negative assortment among mate partners than would be expected at random in the opposite direction as natural-origin pairs) (see Coho Mate Choice Gene Annotations in the supplementary material).

## Discussion

Overall, our results suggest support for the theory of mate choice for heterozygosity in both natural-origin and hatchery-origin mate pairs, at least among SNPs we identified. While positive assortment based on phenotypic characters is relatively common in animals (see Jiang et al. 2013 for a meta-analysis) and assortative mating is evident at the genomic level in humans (Domingue et al. 2014; Robinson et al. 2017), there is also evidence that individuals choose mates based on phenotypic and molecular differences (Brown 1997). In both natural-origin and hatchery-origin mate pairs, there were twice the number of SNPs that trended toward negative assortment compared with positive assortment. This observation of negative assortment is similar to other mate choice studies in salmon (e.g., Landry et al. 2001; see Auld et al. 2019 for a review) and other vertebrates (see Kamiya et al. 2014 for a review), including humans (Havlicek and Roberts 2009) who found mate choice for heterozygosity, particularly at MHC-relevant gene markers.

Although the majority of natural-origin mate pairings showed negative assortment at our identified SNPs, there were SNPs where natural-origin mate pairs demonstrated positive assortment. Notably, there was at least one immune-relevant marker, class I histocompatibility antigen, F10 alpha chain-like, where we did not observe any evidence of mate choice for heterozygosity, but saw assortative mating in natural-origin, but not hatchery-origin mate pairs (see Coho Mate Choice Gene Annotations in the supplementary material). This gene maps to a quantitative trait locus linked to viral resistance, including cardiomyopathy syndrome in Atlantic salmon (Boison et al. 2019). We are unaware of any study that has looked at mate choice with respect to this particular gene, but there is an abundance of evidence that the immune system is important in mate choice (Kamiya et al. 2014).

Although both mating groups demonstrated more negative than positive assortment, SNPs at which natural-origin fish exhibit positive or negative assortment differ from those SNPs at which hatchery-origin fish show negative or positive assortment. It is possible that fish of hatchery-origin make different mate choice decisions based on different exposures and selection pressures experienced by their parents (e.g., captive breeding [Slade et al. 2014]); however, because our experiment draws inference about the nature of mating events (and thus mate choice) solely from evidence we determine from returning offspring genotypes, we draw attention to alternative hypotheses. The “full life-time” aspect of natural selection that transpired before we assayed returning offspring means that there may be other aspects of natural selection, including heterosis, or the positive selection on heterozygous genotypes that is commonly observed in inbred plant and animal lines (e.g., Gemmell and Slate 2006). Both groups of fish being born in the wild would reduce any differences that direct hatchery selection would have, but there would likely remain selective forces in the ocean that could act differently on the offspring of hatchery- or natural-origin fish that were both born in the wild. One potential solution that could enable more direct association of findings to mate choice would be possible if we could genotype embryos soon after fertilization. However, given the threatened status of wild coho in Oregon and the immense interest in wild salmon recovery in the Pacific Northwest, it is unlikely that we would be issued a permit to remove wild embryos from the streams. Additionally, the large total number of embryos we would likely need to sample renders genotyping costs prohibitive given that each female yields ∼3000 eggs. It is also important to note that our experimental design is not able to identify mating pairs that did not produce offspring that returned to their natal spawning grounds. It is possible that both natural-origin and hatchery fish made other mate choice decisions that are not reflected among the offspring that returned to the dam and were sampled. Some spawning events may have resulted in nonviable offspring, offspring that were killed during the fresh or saltwater phase of life, or offspring that strayed (Keefer and Caudill 2014) and thus did not end up being sampled at the weir. In addition, our method of genotyping, GBS, may have missed SNPs that are important in mate choice.

Ours is not the first study to identify a potential role of captive breeding in subsequent mate choice. Slade et al. (2014) observed assortative mating within captive- and wild-bred mice. Any observed differences in mate pairings between hatchery- and natural-origin fish could be the result of transgenerational plasticity in mate choice (Crews et al. 2007; Skinner et al. 2014; Fuxjäjager et al. 2019) from different rearing environments experienced by the previous generation. For example, female rats will avoid mating with males that have a history of exposure to the antiandrogenic fungicide vinclozolin. This avoidance of males with exposure was evident in females three generations removed from the initial exposure of females (i.e., the granddaughters of exposed females) (Crews et al. 2007). This general phenomenon of transgenerational plasticity in mate choice is not limited to mammals, but has also been observed in fish (Fuxjäjager et al. 2019). For example, in stickleback (*Gasterosteus aculeatus*), male mating success is affected by both the temperature of the parental (F0) and offspring's (F1) developmental environment. Specifically, a mismatch in those environments resulted in lower reproductive success of the F1 generation (Fuxjäjager et al. 2019).

Coho salmon use phenotypic traits that may be physical or behavioral when choosing their mates (see Auld et al. 2019 for a review). Whether any of these traits are associated with the SNPs identified in our study remains unknown. It is also possible that the genetic markers we identified are not responsible for any trait that may be used for mate selection, but that those genetic markers are linked to other genes that are responsible for the expression of traits that are relevant for sexual selection. Further insight into the phenotypic traits associated with these genetic markers is possible with gene editing technologies, such as CRISPR/Cas9.

Understanding mate choice in captive-bred species has been identified as important for establishing and maintaining successful conservation and captive breeding programs (Greggor et al. 2016). Because mate choice decisions can be plastic and vary according to current environmental conditions, including abiotic and biotic factors, it is important to determine whether findings are generalizable across space and time or whether genetic mating patterns differ between populations, environmental conditions, and/or over time. Future comparisons with other populations will enable identification of any generalizable trends. Our study provides discrete evidence from a specific group of SNPs, one species, and context. Greater knowledge obtained from additional studies on the genetic basis of mate choice will enable better overall understanding of mate choice and its importance in evolution and genetic patterns found throughout nature.

## Supplementary Material

obab020_Supplemental_Files

## Data Availability

Data used in this study is available at Oregon State University's Scholars Archive https://doi.org/10.7267/N9H41PB9_v2.
