# Supplementary material for: Differences in Mate Pairings of Hatchery- and Natural-Origin Coho Salmon Inferred from Offspring Genotypes
Source: Integr Org Biol. 2021 Aug 14;3(1):obab020. doi: 10.1093/iob/obab020 (PMC8363981; doi:10.1093/iob/obab020)
Supplement: obab020_Supplemental_Files [file obab020_supplemental_files.zip › ESM 1 Coho Mate Choice Figure S1.pdf]

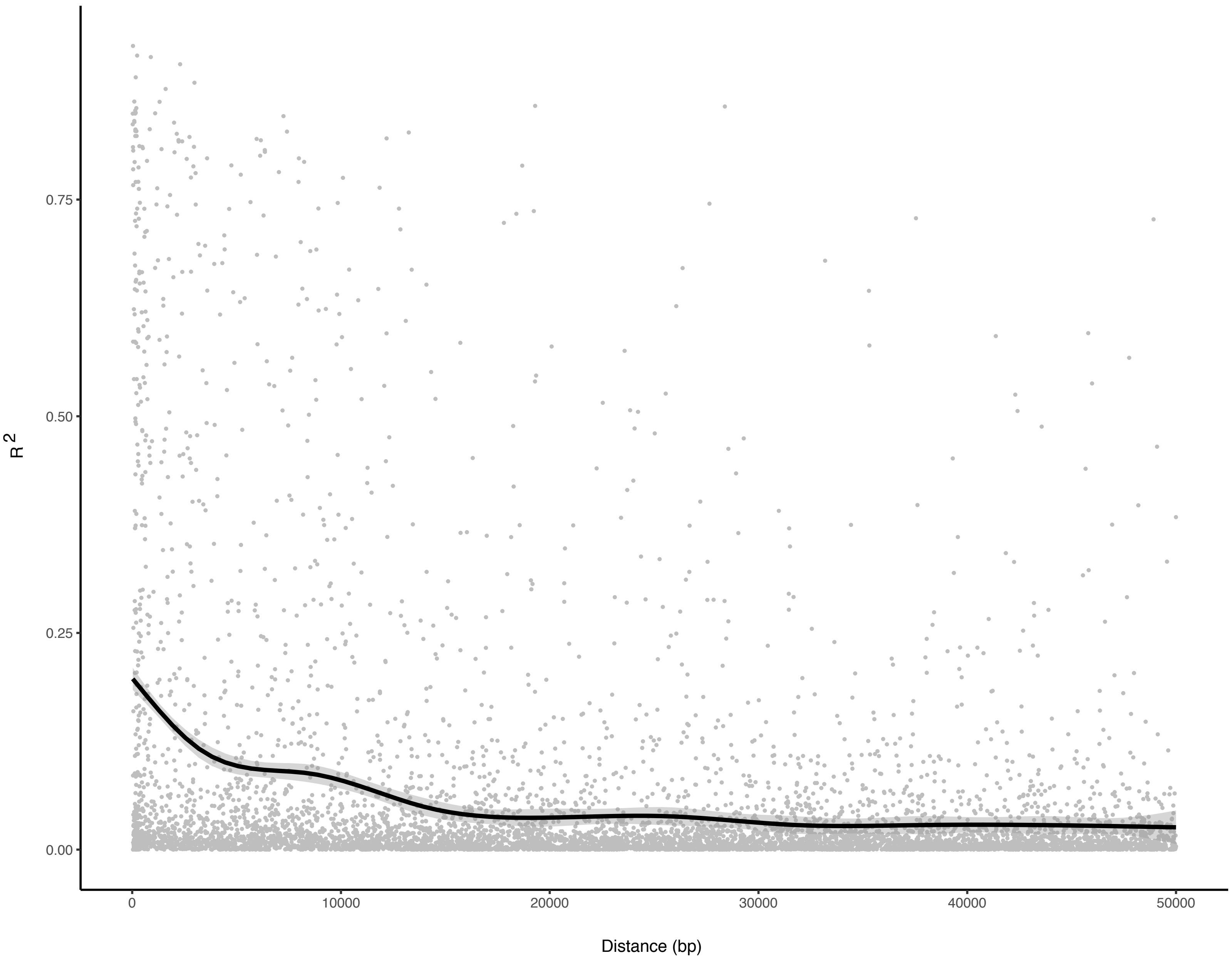


**Figure S2**. Linkage disequilibrium across the coho genome given as R2 values relative to distance (bp) between loci.
