# Supplementary material for: Differences in Mate Pairings of Hatchery- and Natural-Origin Coho Salmon Inferred from Offspring Genotypes
Source: Integr Org Biol. 2021 Aug 14;3(1):obab020. doi: 10.1093/iob/obab020 (PMC8363981; doi:10.1093/iob/obab020)
Supplement: obab020_Supplemental_Files [file obab020_supplemental_files.zip › ESM 2 Coho Mate Choice Code.docx]

**Coho mate choice - Code for analysis**

#process radtags

nano procrad.txt

process_radtags -i gzfastq -1 lane1-s001-index--GBS0125_S1_L001_R1_001.

fastq.gz -2 lane1-s001-index--GBS0125_S1_L001_R2_001.fastq.gz -o procrad

-b Barcodes_GBS0125.txt -c --renz_1 pstI --renz_2 mspI -q -t 134 -E phre

d33 --adapter_1 ACACTCTTTCCCTACACGACGCTCTTCCGATCT --adapter_2 AGATCGGAAG

AGCGTCGTGTAGGGAAAGAGTGT --adapter_mm 2

process_radtags -i gzfastq -1 lane2-s002-index--GBS0126_S2_L002_R1_001.

fastq.gz -2 lane2-s002-index--GBS0126_S2_L002_R2_001.fastq.gz -o procrad

-b Barcodes_GBS0126.txt -c --renz_1 pstI --renz_2 mspI -q -t 134 -E phre

d33 --adapter_1 ACACTCTTTCCCTACACGACGCTCTTCCGATCT --adapter_2 AGATCGGAAG

AGCGTCGTGTAGGGAAAGAGTGT --adapter_mm 2

process_radtags -i gzfastq -1 lane3-s003-index--GBS0127_S3_L003_R1_001.

fastq.gz -2 lane3-s003-index--GBS0127_S3_L003_R2_001.fastq.gz -o procrad

-b Barcodes_GBS0127.txt -c --renz_1 pstI --renz_2 mspI -q -t 134 -E phre

d33 --adapter_1 ACACTCTTTCCCTACACGACGCTCTTCCGATCT --adapter_2 AGATCGGAAG

AGCGTCGTGTAGGGAAAGAGTGT --adapter_mm 2

process_radtags -i gzfastq -1 lane4-s004-index--GBS0128_S4_L004_R1_001.

fastq.gz -2 lane4-s004-index--GBS0128_S4_L004_R2_001.fastq.gz -o procrad

-b Barcodes_GBS0128.txt -c --renz_1 pstI --renz_2 mspI -q -t 134 -E phre

d33 --adapter_1 ACACTCTTTCCCTACACGACGCTCTTCCGATCT --adapter_2 AGATCGGAAG

AGCGTCGTGTAGGGAAAGAGTGT --adapter_mm 2

process_radtags -i gzfastq -1 lane5-s005-index--GBS0129_S5_L005_R1_001.

fastq.gz -2 lane5-s005-index--GBS0129_S5_L005_R2_001.fastq.gz -o procrad

-b Barcodes_GBS0129.txt -c --renz_1 pstI --renz_2 mspI -q -t 134 -E phre

d33 --adapter_1 ACACTCTTTCCCTACACGACGCTCTTCCGATCT --adapter_2 AGATCGGAAG

AGCGTCGTGTAGGGAAAGAGTGT --adapter_mm 2

process_radtags -i gzfastq -1 lane6-s006-index--GBS0130_S6_L006_R1_001.

fastq.gz -2 lane6-s006-index--GBS0130_S6_L006_R2_001.fastq.gz -o procrad

-b Barcodes_GBS0130.txt -c --renz_1 pstI --renz_2 mspI -q -t 134 -E phre

d33 --adapter_1 ACACTCTTTCCCTACACGACGCTCTTCCGATCT --adapter_2 AGATCGGAAG

AGCGTCGTGTAGGGAAAGAGTGT --adapter_mm 2

process_radtags -i gzfastq -1 lane7-s007-index--GBS0131_S7_L007_R1_001.

fastq.gz -2 lane7-s007-index--GBS0131_S7_L007_R2_001.fastq.gz -o procrad

-b Barcodes_GBS0131.txt -c --renz_1 pstI --renz_2 mspI -q -t 134 -E phre

d33 --adapter_1 ACACTCTTTCCCTACACGACGCTCTTCCGATCT --adapter_2 AGATCGGAAG

AGCGTCGTGTAGGGAAAGAGTGT --adapter_mm 2

process_radtags -i gzfastq -1 lane8-s008-index--GBS0132_S8_L008_R1_001.

fastq.gz -2 lane8-s008-index--GBS0132_S8_L008_R2_001.fastq.gz -o procrad

-b Barcodes_GBS0132.txt -c --renz_1 pstI --renz_2 mspI -q -t 134 -E phre

d33 --adapter_1 ACACTCTTTCCCTACACGACGCTCTTCCGATCT --adapter_2 AGATCGGAAG

AGCGTCGTGTAGGGAAAGAGTGT --adapter_mm 2

process_radtags -i gzfastq -1 lane1-s001-index--GBS0133_S1_L001_R1_001.

fastq.gz -2 lane1-s001-index--GBS0133_S1_L001_R2_001.fastq.gz -o procrad

-b Barcodes_GBS0133.txt -c --renz_1 pstI --renz_2 mspI -q -t 134 -E phre

d33 --adapter_1 ACACTCTTTCCCTACACGACGCTCTTCCGATCT --adapter_2 AGATCGGAAG

AGCGTCGTGTAGGGAAAGAGTGT --adapter_mm 2

#create file for bwa commands

nano bwacommands.sh

for i in procrad/*.fq.gz;

do

if ! [[( $i == *"rem"*) || ($i == *".2.fq"*)]];

then

*# echo "contains rem"*

*#else*

*# echo $i*

newfile=$(basename $i .1.fq.gz)

*# echo $newfile*

echo "bwa mem -M -t 4 GCF_002021735.1_Okis_V1_genomic.fa $i proc

rad/${newfile}.2.fq.gz > samfiles/${newfile}.sam"

fi

done

#create bwa commands

sh bwacommands.sh > bwacommands.txt

#run bwa commands

cat bwacommands.txt | SGE_Array -P 1 -b 16 -r BWALOG -q harold -m 32G

#sort sam and convert to bam

nano sortsamtobam.sh

for i in samfiles/*.sam;

do

newfile=$(basename $i .sam)

echo "samtools view -b $i.sam | samtools sort - threads > $i.bam"

done

#create sort sam commands

sh sortsamtobam.sh > sortsamtobam.txt

#run sam to bam and sort

cat sortsamtobam.txt | SGE_Array -P 1 -b 16 -r SORTLOG -q harold -m 32G

#commands to check reads in bam files

nano Bam_ReadCount.sh

for i in bamfiles/*.bam;

do

echo "samtools view -c $i"

done

#create read count command

sh Bam_ReadCount.sh > Bam_ReadCount.txt

#run Bam_ReadCount

cat Bam_ReadCount.txt | SGE_Array -P 1 -b 16 -r BAMCOUNTLOG -q harold

#remove small bam files from population map (done by hand)

#run stacks and populations

/stacks-2.41/bin/ref_map.pl -T 30 -o /stacks.ref2 --popmap cohopopmapnos

mallbam.txt --samples /bamfiles -X "populations: -r 0.8 -p 1 --min_maf

0.05 --write_single_snp --structure --fstats --fst_correction bonferron

i_win --genepop --plink --vcf --fasta_loci --fasta_samples" -r coho.p1r8

#run paralog finder for Blacklist

tcsh

../paralog-finder/HDplot_process_vcf.py -i populations.snps.vcf

bash

../paralog-finder/HDplot_graphs.R -i populations.depthsBias --minD -80 -

-maxD 80

tcsh

#select D values based on graph outputs

../paralog-finder/blacklist_paralogs.py -i populations.depthsBias --maxH

0.6 --minD -5 --maxD 7

#modify format of blacklist

sed 's/\:.*//' populations_paralogs.blacklist > populations_paralogs.bla

cklist

#run populations with Blacklist from paralog finder

/stacks-2.41/bin/populations -t 30 -O /stacks.ref2BL --popmap cohopopmap

nosmallbam.txt -P /stacks.ref2 -r 0.8 -p 1 --min_maf 0.05 --blacklist st

acks.ref2/populations_paralogs.blacklist --write_single_snp --structure

--fstats --fst_correction bonferroni_win --genepop --plink --vcf --fasta

_loci --fasta_samples

#remove individuals missing more than 20% of sites

vcftools --vcf /stacks.p1r8BL/populations.snps.vcf --missing-indv

awk '$5 > 0.8' out.imiss | cut -f1 > lowDP.indv

vcftools --vcf populations.snps.vcf --remove lowDP.indv --recode --recod

e-INFO-all --out populations.snps.removeLI

#remove sites with mean depth less than 5 and greater than 30

vcftools --vcf /stacks.p1r8BL/populations.snps.removeLI.recode.vcf --min

-meanDP 5 --max-meanDP 30 --recode --out populations.snps.removeLI.MMDP5

30

#remove sites with max missing over 20%

vcftools --vcf /stacks.p1r8BL/populations.snps.removeLI.MMDP530.recode.v

cf --max-missing .8 --recode --out populations.snps.removeLI.MMDP530.MM8

#remove sites out of HWE

vcftools --vcf /stacks.p1r8BL/populations.snps.removeLI.MMDP530.MM8.reco

de.vcf --hwe --recode --out populations.snps.removeLI.MMDP530.MM8HWE

#calculate depth, site depth, HWE statistics

vcftools --vcf /stacks.p1r8BL/populations.snps.removeLI.MMDP530.MM8.reco

de.vcf --depth

vcftools --vcf /stacks.p1r8BL/populations.snps.removeLI.MMDP530.MM8.reco

de.vcf --site-depth

vcftools --vcf /stacks.p1r8BL/populations.snps.removeLI.MMDP530.MM8.reco

de.vcf --hardy

#calculate miscall rate using whoa in R / ESM 1 Figure S1

#load programs

library(whoa)

library(vcfR)

#load file

coho<-vcfR::read.vcfR("/stacks.p1r8BL/populations.snps.removeLI.MMDP530.

MM8.recode.vcf")

#compute expected and observed genotype frequencies

gfreqs <- exp_and_obs_geno_freqs(coho)

#plot those. Set max plot loci so that all loci are plotted

geno_freqs_scatter(gfreqs, max_plot_loci = 20000)

#calculate overall hetero miscall rate

overall <- infer_m(coho, minBin = 1e15)

overall$m_posteriors

binned <- infer_m(coho, minBin = 2000)

posteriors_plot(binned$m_posteriors)

#convert filtered vcf file to plink format for LD analysis

plink --vcf populations.snps.removeLI.MMDP530.MM8.recode.vcf --recode --

out populations.snps.removeLI.MMDP530.MM8.recode.vcf --allow-extra-chr

#linkage disequilibrium in plink/ ESM 2 Figure S2

plink --file populations.snps.removeLI.MMDP530.MM8.recode.vcf --r2 --ldwindow-r2 0 --ld-window 100000 --ld-window-kb 100 --allow-extra-chr --out populations.snps.removeLI.MMDP530.MM8.recode.vcf

#calculate number of SNPs per chromosome

vcftools --vcf populations.snps.removeLI.MMDP530.MM8.recode.vcf --freq -

-chr NC_034174.1 --out output1

vcftools --vcf populations.snps.removeLI.MMDP530.MM8.recode.vcf --freq -

-chr NC_034175.1 --out output2

vcftools --vcf populations.snps.removeLI.MMDP530.MM8.recode.vcf --freq -

-chr NC_034176.1 --out output3

vcftools --vcf populations.snps.removeLI.MMDP530.MM8.recode.vcf --freq -

-chr NC_034177.1 --out output4

vcftools --vcf populations.snps.removeLI.MMDP530.MM8.recode.vcf --freq -

-chr NC_034178.1 --out output5

vcftools --vcf populations.snps.removeLI.MMDP530.MM8.recode.vcf --freq -

-chr NC_034179.1 --out output6

vcftools --vcf populations.snps.removeLI.MMDP530.MM8.recode.vcf --freq -

-chr NC_034180.1 --out output7

vcftools --vcf populations.snps.removeLI.MMDP530.MM8.recode.vcf --freq -

-chr NC_034181.1 --out output8

vcftools --vcf populations.snps.removeLI.MMDP530.MM8.recode.vcf --freq -

-chr NC_034182.1 --out output9

vcftools --vcf populations.snps.removeLI.MMDP530.MM8.recode.vcf --freq -

-chr NC_034183.1 --out output10

vcftools --vcf populations.snps.removeLI.MMDP530.MM8.recode.vcf --freq -

-chr NC_034184.1 --out output11

vcftools --vcf populations.snps.removeLI.MMDP530.MM8.recode.vcf --freq -

-chr NC_034185.1 --out output12

vcftools --vcf populations.snps.removeLI.MMDP530.MM8.recode.vcf --freq -

-chr NC_034186.1 --out output13

vcftools --vcf populations.snps.removeLI.MMDP530.MM8.recode.vcf --freq -

-chr NC_034187.1 --out output14

vcftools --vcf populations.snps.removeLI.MMDP530.MM8.recode.vcf --freq -

-chr NC_034188.1 --out output15

vcftools --vcf populations.snps.removeLI.MMDP530.MM8.recode.vcf --freq -

-chr NC_034189.1 --out output16

vcftools --vcf populations.snps.removeLI.MMDP530.MM8.recode.vcf --freq -

-chr NC_034190.1 --out output17

vcftools --vcf populations.snps.removeLI.MMDP530.MM8.recode.vcf --freq -

-chr NC_034191.1 --out output18

vcftools --vcf populations.snps.removeLI.MMDP530.MM8.recode.vcf --freq -

-chr NC_034192.1 --out output19

vcftools --vcf populations.snps.removeLI.MMDP530.MM8.recode.vcf --freq -

-chr NC_034193.1 --out output20

vcftools --vcf populations.snps.removeLI.MMDP530.MM8.recode.vcf --freq -

-chr NC_034194.1 --out output21

vcftools --vcf populations.snps.removeLI.MMDP530.MM8.recode.vcf --freq -

-chr NC_034195.1 --out output22

vcftools --vcf populations.snps.removeLI.MMDP530.MM8.recode.vcf --freq -

-chr NC_034196.1 --out output23

vcftools --vcf populations.snps.removeLI.MMDP530.MM8.recode.vcf --freq -

-chr NC_034197.1 --out output24

vcftools --vcf populations.snps.removeLI.MMDP530.MM8.recode.vcf --freq -

-chr NC_034198.1 --out output25

vcftools --vcf populations.snps.removeLI.MMDP530.MM8.recode.vcf --freq -

-chr NC_034199.1 --out output26

vcftools --vcf populations.snps.removeLI.MMDP530.MM8.recode.vcf --freq -

-chr NC_034200.1 --out output27

vcftools --vcf populations.snps.removeLI.MMDP530.MM8.recode.vcf --freq -

-chr NC_034201.1 --out output28

vcftools --vcf populations.snps.removeLI.MMDP530.MM8.recode.vcf --freq -

-chr NC_034202.1 --out output29

vcftools --vcf populations.snps.removeLI.MMDP530.MM8.recode.vcf --freq -

-chr NC_034203.1 --out output30

#plot LD in R

#load packages

library(ggplot2)

#load data

ld <- read.table("/stacks.p1r8BL/populations.snps.removeLI.MMDP530.MM8.r

ecode.vcf.ld",header=T)

attach(ld)

#plot the average correlation for each snp distance

ggplot(ld, aes(x=BP_B - BP_A, y = R2)) +

geom_point(colour = "grey", size =0.5) +

geom_smooth(aes(x=BP_B - BP_A, y = R2), colour ="black") +

theme(panel.grid.major = element_blank(), panel.grid.minor = element_b

lank(),

panel.background = element_blank(), axis.line = element

_line(colour = "black")

###convert vcf file to numerical genotypes in Tassel GUI

###convert 0, 0.5, 1 genotypes to 0,1,2 in excel

#format file for permuatations (clean up) in R

DataRecode<-read.table("/stacks.p1r8BL/Coho_revised_numeric.txt", header

= TRUE)

DataM<-read.table("2005MatePair_Stacks_onepop_noparalogMALE.txt", header

= TRUE)

DataF<-read.table("2005MatePair_Stacks_onepop_noparalogFEMALE.txt", head

er = TRUE)

DataMale<-merge(DataM, DataRecode, by = "ID")

DataFemale<-merge(DataF, DataRecode, by ="ID")

DataMF<-merge(DataMale, DataFemale, by = "PAIR")

SNPONLY<-subset(DataMF, select=-c(ID.y, TACTIC.y, HORW_M.y, HORW_F.y, MA

LE))

SNPONLY<-rename(SNPONLY, replace = c("ID.x" = "MALE"))

orig_df<-SNPONLY

#delete INDEX column and PAIR column

orig_df$INDEX <- NULL

orig_df$PAIR <- NULL

orig_df$TACTIC.x<- NULL

orig_df$HORW_M.x<- NULL

orig_df$HORW_F.x<- NULL

#.x columns are male genotypes and .y female; rename them

colnames(orig_df) <- str_replace(colnames(orig_df), ".x$", "@MALE_GENO")

colnames(orig_df) <- str_replace(colnames(orig_df), ".y$", "@FEMALE_GEN

O")

#make it long

long <- gather(orig_df, LOCUS, GENOTYPE, -FEMALE, -MALE)

#split e.g. S1_56921@MALE into two cols, S1_56921 and MALE

long_split <- extract(long, LOCUS, c("LOCUS", "SEX"), regex = "(.+)@(.

+)")

#make genotype cols for male and female seperately

rewide <- spread(long_split, SEX, GENOTYPE)

coho_stacks_revisions_long <- rewide

save(coho_stacks_revisions_long, file = "coho_stacks_long.rdat")

#Permutations

*#!/usr/bin/env Rscript*

suppressPackageStartupMessages(library(dplyr))

suppressPackageStartupMessages(library(tidyr))

suppressPackageStartupMessages(library(stringr))

suppressPackageStartupMessages(library(parallel))

suppressPackageStartupMessages(library(purrr))

NUM_CPUS <- 48

NUM_PERMUTATIONS <- 50000

load("/stacks.p1r8BL/coho_stacks_long.rdat")

groups_data <- read.table("/Stacks/Coho_Stacks_MatePairs_Origin.txt", header = T, sep = "\t", stringsAsFactors = F)

wild_males <- unique(groups_data$MALE[groups_data$ORIGIN_MALE == "W"])

hatchery_males <- unique(groups_data$MALE[groups_data$ORIGIN_MALE == "H"])

wild_females <- unique(groups_data$FEMALE[groups_data$ORIGIN_FEMALE =="W"])

hatchery_females <- unique(groups_data$FEMALE[groups_data$ORIGIN_FEMALE == "H"])

geno_data_wild_wild <- coho_stacks_revisions_long[coho_stacks_revisions_long$MALE %in% wild_males &

coho_stacks_revisions_long$FEMALE %in% wild_females, ]

geno_data_hatchery_hatchery <- coho_stacks_revisions_long[coho_stacks_revisions_long$MALE %in% hatchery_males &

coho_stacks_revisions_long$FEMALE %in% hatchery_females, ]

geno_data_wild_hatchery <- coho_stacks_revisions_long[coho_stacks_revisions_long$MALE %in% wild_males &

coho_stacks_revisions_long$FEMALE %in% hatchery_females, ]

geno_data_hatchery_wild <- coho_stacks_revisions_long[coho_stacks_revisions_long$MALE %in% hatchery_males &

coho_stacks_revisions_long$FEMALE %in% wild_females, ]

geno_data_w_h_or_h_w <- rbind(geno_data_wild_hatchery, geno_data_hatchery_wild)

# just to remember what we're looking at

print(head(coho_stacks_revisions_long))

#Given a df with cols MALE_GENO and FEMALE_GENO, where 0 means 0 minor alleles, 1 means 1 minor allele, and 2 means 2 minor alleles, adds a SHARED_AL LELES column indicating how many alleles are shared between MALE_GENO and FEMALE_GENO

add_shared_alleles_column <- function(df) {

df$SHARED_ALLELES <- NA

#if neither are NA, we'll start by assuming there are no shared alleles

df$SHARED_ALLELES[!is.na(df$FEMALE_GENO) &

!is.na(df$MALE_GENO)] <- 0

#if the genotype is equal, there are 2 shared alleles

df$SHARED_ALLELES[df$FEMALE_GENO ==

df$MALE_GENO &

!is.na(df$FEMALE_GENO) &

!is.na(df$MALE_GENO)] <- 2

#if they are not equal, and one of them is a 1, there is 1 shared allele

df$SHARED_ALLELES[df$FEMALE_GENO !=

df$MALE_GENO &

!is.na(df$FEMALE_GENO) &

!is.na(df$MALE_GENO) &

(df$FEMALE_GENO == 1 |

df$MALE_GENO == 1)] <- 1

return(df)

}

#given a dataframe with columns for LOCUS, MALE (id), FEMALE (id) (a mating for this locus0, MALE_GENO and FEMALE_GENO, extracts just the data for locus_to_test,and randomizes the male information num_permuation times, computing the mean shared alleles and sd shared alleles each time.

#Looks at the original mean and sd of shared alleles, and computes thepercentile of those across the permuations.

#Returns a single-row data frame of this information

permutation_test_for_locus2 <- function(locus_to_test, df, num_permutati

ons) {

orig_locus <- df[df$LOCUS == locus_to_test, ]

orig_locus <- add_shared_alleles_column(orig_locus)

orig_mean_shared_alleles <- mean(orig_locus$SHARED_ALLELES, na.rm = T)

orig_sd_shared_alleles <- sd(orig_locus$SHARED_ALLELES, na.rm = T)

mean_shared_alleles_vec <- rep(NA, num_permutations)

sd_shared_alleles_vec <- rep(NA, num_permutations)

male_df <- orig_locus[, c("MALE", "MALE_GENO")]

#shuffle the females

for(permutation_number in seq(1, num_permutations)) {

female_df <- orig_locus[sample(seq(1, nrow(orig_locus))), c("FEMALE"

, "FEMALE_GENO")]

rejoined <- cbind(male_df, female_df)

rejoined <- add_shared_alleles_column(rejoined)

mean_shared <- mean(rejoined$SHARED_ALLELES, na.rm = T)

sd_shared <- sd(rejoined$SHARED_ALLELES, na.rm = T)

mean_shared_alleles_vec[permutation_number] <- mean_shared

sd_shared_alleles_vec[permutation_number] <- sd_shared

}

mean_percentile <- length(mean_shared_alleles_vec[orig_mean_shared_alleles >

mean_shared_alleles_vec])/length(mean_shared_alleles_vec)

sd_percentile <- length(sd_shared_alleles_vec[orig_sd_shared_alleles >

sd_shared_alleles_vec])/length(sd_shared_alleles_vec)

mean_of_means <- mean(mean_shared_alleles_vec)

sd_of_means <- sd(mean_shared_alleles_vec)

mean_of_sds <- mean(sd_shared_alleles_vec)

sd_of_sds <- sd(sd_shared_alleles_vec)

return(data.frame(mean_percentile = mean_percentile, sd_percentile = s

d_percentile, locus = locus_to_test,

mean_of_means = mean_of_means, sd_of_means = sd_of_m

eans, mean_of_sds = mean_of_sds, sd_of_sds = sd_of_sds,

orig_mean_shared_alleles = orig_mean_shared_alleles,

orig_sd_shared_alleles = orig_sd_shared_alleles))

}

#make lapply() run in parallel across NUM_CPUS

cl <- makeCluster(NUM_CPUS)

clusterExport(cl, "add_shared_alleles_column")

lapply <- function(...) {parLapply(cl, ...)}

#get a list of loci to work over for lapply

loci_list <- as.list(unique(coho_stacks_revisions_long$LOCUS))

#compute the results in parallel

system.time(results_list <- lapply(loci_list, permutation_test_for_locus

2, geno_data_wild_wild, NUM_PERMUTATIONS))

#turn the resulting list of data frames into a single data frame

results_table <- map_df(results_list, I)

#save it

write.table(results_table,

"wildm_wildf_coho_stacks_loci_percentiles.txt",

quote = F,

sep = "\t",

row.names = F)

#compute the results in parallel

system.time(results_list <- lapply(loci_list, permutation_test_for_locus

2, geno_data_hatchery_hatchery, NUM_PERMUTATIONS))

#turn the resulting list of data frames into a single data frame

results_table <- map_df(results_list, I)

#save it

write.table(results_table,

"hatcherym_hatcheryf_coho_stacks_loci_percentiles.txt",

quote = F,

sep = "\t",

row.names = F)

#figure 2

#load data

data <- read.table("Coho_Sig_Figure.txt", header = TRUE)

#load library

library(ggplot2)

#panel A

data$Chromsome1<-factor(data$Chromsome1, levels = c("1", "2", "3", "4",

"5", "6", "7", "8",

"9", "10", "11", "12",

"13", "14", "15", "16",

"17", "18", "19", "20",

"21", "22", "23", "24",

"25", "26", "27", "28",

"29", "30", "Unplaced"))

ggplot(data, aes(fill=Category1, y=Count1, x=Chromsome1)) +

geom_bar(position="stack", stat="identity") +

theme(panel.grid.major = element_blank(), panel.grid.minor = element_blank(),

panel.background = element_blank(), axis.line = element_line(colour = "black"),

axis.text.x = element_text(angle = 90)) + scale_fill_manual(values=cbPalette)

#panel b

data$Chromsome2<-factor(data$Chromsome2, levels = c("1", "2", "3", "4",

"5", "6", "7", "8",

"9", "10", "11", "12",

"13", "14", "15", "16",

"17", "18", "19", "20",

"21", "22", "23", "24",

"25", "26", "27", "28",

"29", "30", "Unplaced"))

ggplot(data, aes(fill=Category2, y=Count2, x=Chromsome2)) +

geom_bar(position="stack", stat="identity") +

theme(panel.grid.major = element_blank(), panel.grid.minor = element_blank(),

panel.background = element_blank(), axis.line = element_line(colour = "black"),

axis.text.x = element_text(angle = 90)) + scale_fill_manual(values=cbPalette)

**REFERENCES**

Henry, L., Wickham, H. (2018). purrr: Functional Programming Tools. R

package version 0.2.5. <https://CRAN.R-project.org/package=purrr>

R Core Team (2018) R: A language and environment for statistical computing. R Foundation for Statistical Computing, Vienna, Austria.

Wickham, H., François, R., Henry, L., Müller, K. (2018). Dplyr: A grammar of data manipulation. R package version 0.7.5. <https://CRAN.R-project.org/package=dplyr>

Wickham, H., Henry, L. (2018). tidyr: Easily Tidy Data with 'spread()' and 'gather()' Functions. R package version 0.8.1. https://CRAN.R-project.org/package=tidyr
