# Supplementary material for: Differences in Mate Pairings of Hatchery- and Natural-Origin Coho Salmon Inferred from Offspring Genotypes
Source: Integr Org Biol. 2021 Aug 14;3(1):obab020. doi: 10.1093/iob/obab020 (PMC8363981; doi:10.1093/iob/obab020)
Supplement: obab020_Supplemental_Files [file obab020_supplemental_files.zip › ESM 3 Coho Mate Choice Figure S2.pdf]

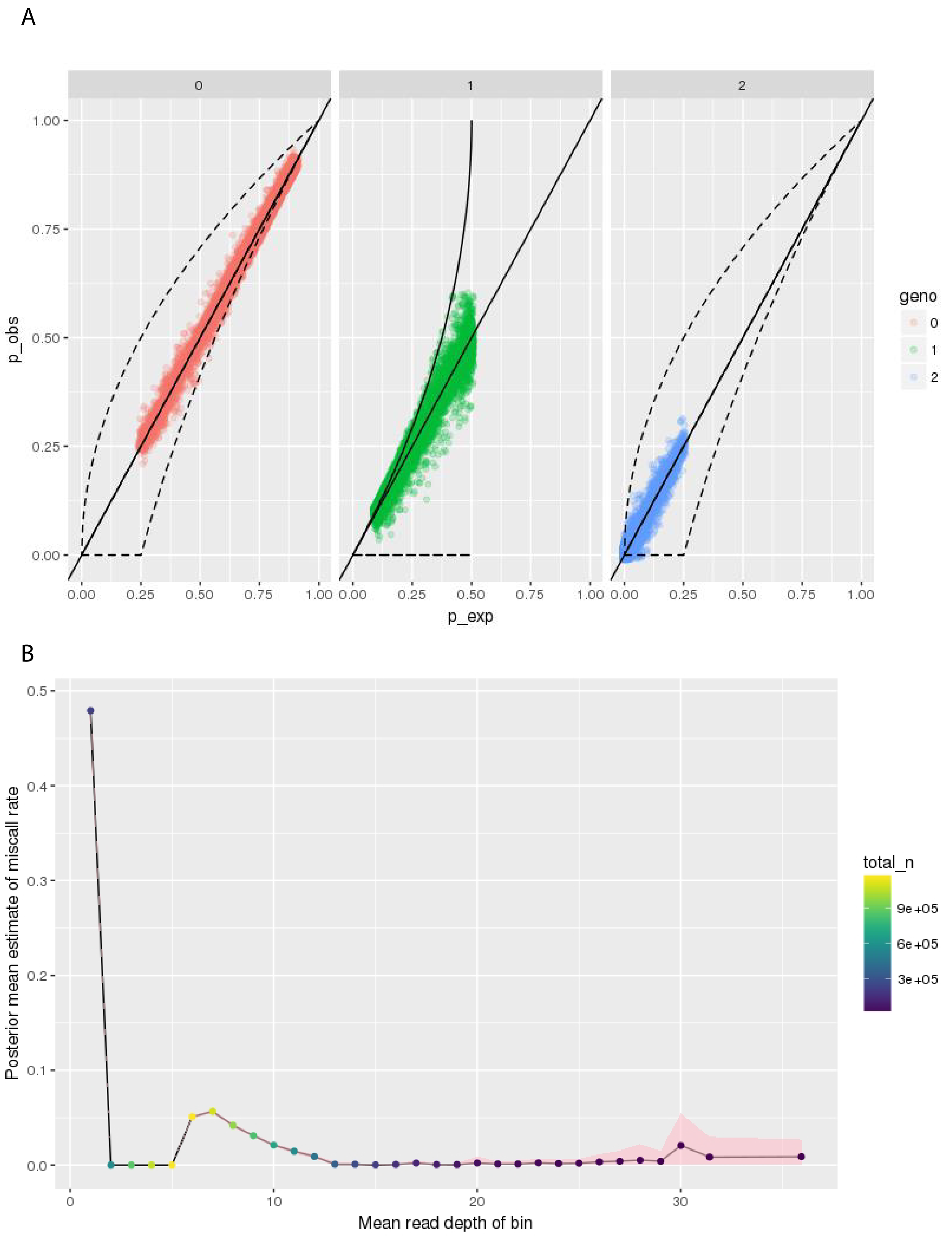

Figure 2. A) Scatterplot of observed and expected genotypes where 0 is homozygous for the major allele, 1 is heterozygous and 2 is homozygous for the minor allele. B) Posterior mean estimate of miscall rate for mean read depth.
